# 

**Published:** 1842-02

**Authors:** 


### CONTENTS

OF NO. II.

### ORIGINAL COMMUNICATIONS.

### ESSAYS AND CASES.

Art. I.—What shall we do with our Insane? By Edward Jarvis, M. D., Louisville, Ky. - - 81
Art. II.—Notes on the Scarlet Fever, as it appeared in Green County, Ohio. By John Dawson, M. D. - 126

### REVIEWS.

Art. III.—The Philosophy of Storms. By James P. Espy, A. M., Member of the American Philosophical Society, and Corresponding Member of the National Institution, Washington. Boston: Charles C. Little and James Brown. 1841. pp. 552. - - 129

### BIBLIOGRAPHICAL NOTICES.

Art. IV.—The Guardian of Health; a monthly Journal of Domestic Hygiene. - - - - - 139
Art. V.—A Report of the Facts and Circumstances relating to a case of Compound Fracture and Prosecution for Mal-Practice, in which William Smith was Plaintiff, and Drs. Goodyear and Hyde Defendants, at Cortland

Village, Cortland County, N. Y. March, 1841: Comprising statements of the case by several medical gentlemen, together with notes and comments on the Testimony. By A. B. Shipman, M. D. Cortlandville, 1841. pp. 35. - - - - 141

SELECTIONS FROM AMERICAN AND FOREIGN JOURNALS.

Case of Deformed Leg, from unsuccessfully treated Fracture, cured by an operation performed by John Rhea Barton, M. D. Reported by W. S. W. Ruschenberger, M. D., U. S. Navy. - - - - 149

ORIGINAL INTELLIGENCE.

An Explanation. - - - - - 157

Digitalis. - - - - - - 157

Calomel reduced to the protoxide in the Primæ Viæ. - - 159

Lithotomy—again. - - - - - 159

Polypus Uteri. - - - - - - 159

